# Plasmids of the multidrug-resistant *Citrobacter portucalensis* KOS1-1 strain isolated from a wastewater treatment plant harbor antibiotic resistance genes and gene clusters involved in carbon metabolism

**DOI:** 10.1128/spectrum.02038-25

**Published:** 2026-01-14

**Authors:** Shahjahon Begmatov, Andrey L. Rakitin, Alexey V. Beletsky, Andrey V. Mardanov, Nikolai V. Ravin

**Affiliations:** 1 Institute of Bioengineering, Research Center of Biotechnology of the Russian Academy of Sciences 54744, Moscow, Russia; Universidad Maimonides, Buenos Aires, Argentina

**Keywords:** plasmid, multidrug-resistant bacteria, cellulose biosynthesis, glycerol dissimilation

## Abstract

The growing interest in multidrug-resistant (MDR) *Citrobacter* species stems from their epidemiological significance and their potential to harbor antibiotic resistance genes (ARGs), with mobile genetic elements playing a central role in their dissemination. Wastewater treatment plants play an important role in the formation of such MDR strains due to the high rate of horizontal gene transfer in these environments. In this study, we isolated the MDR strain KOS1-1 of *Citrobacter portucalensis* from wastewater, sequenced its genome, and characterized its ARG content and plasmid profile. This strain was resistant to ampicillin, cefazolin, cefaclor, cefatrizine, ciprofloxacin, kanamycin, streptomycin, spectinomycin, erythromycin, chloramphenicol, tetracycline, sulfamethoxazole, and trimethoprim. The KOS1-1 strain harbored five low copy number plasmids ranging in size from 77,569 to 289,033 bp. Genome analysis revealed the presence of multiple ARGs both on the chromosome and on plasmids, conferring resistance to β-lactams, quinolones, aminoglycosides, macrolides, sulfonamides, trimethoprim, phenicols, and tetracyclines. Many of these genes were associated with pseudo-composite transposon-like structures, emphasizing the role of mobile elements in ARGs dissemination. Plasmids harbored a bacterial cellulose biosynthesis operon and genes involved in mannose/fucose metabolism that could facilitate biofilm formation and a glycerol dissimilation gene cluster. Bacterial cellulose production was confirmed using electron and atomic force microscopy. Homologus gene clusters were identified on various plasmids of *Enterobacteriales*, suggesting their distribution via horizontal gene transfer. The presence of plasmids carrying ARGs and adaptive accessory genes increases the competitive fitness of *C. portucalensis* KOS1-1.

**IMPORTANCE:**

Antimicrobial resistance represents a silent epidemic that has emerged as a critical global concern in recent years, underscoring the need for further research in this field. This study aimed to isolate and characterize multidrug-resistant bacteria from municipal wastewater, a huge reservoir of antibiotic resistance genes and resistant strains, from which they became disseminated into the environment. The isolated *Citrobacter portucalensis* strain KOS1-1 exhibits resistance to multiple antibiotics, arsenate, and mercury. It harbors five megaplasmids containing most of the resistance genes, along with laterally acquired bacterial cellulose biosynthesis operon and genes associated with mannose/fucose metabolism, which may facilitate biofilm formation. These plasmids may not only confer a selective advantage to host strains but also promote transfer of resistance determinants in high-density microbial communities of activated sludge at wastewater treatment plants. This work contributes to the understanding of the mechanisms of dissemination of bacterial resistance and virulence factors in municipal wastewater environments.

## INTRODUCTION

Multidrug-resistant (MDR) bacteria are formed through the acquisition of genetic determinants of resistance under the influence of anthropogenic factors. Wastewater treatment plants (WWTPs) can serve as an “ideal” system for the formation of such MDR strains due to the high rate of horizontal gene transfer (1). Therefore, such MDR bacteria and resistance genes can enter the environment with purified water, spread in aquatic ecosystems and soils, and be transmitted to other bacterial strains. Subsequently, they can “return” to humans, for example, through water supplies or agricultural products, so it is important to regularly monitor the spread of antibiotic resistance not only in the healthcare system but also in the environment.

The importance of isolating and culturing multidrug-resistant bacteria from WWTP samples (raw sewage, activated sludge, and treated effluent) is that it may provide insights not only into the genetic determinants of resistance but also into the metabolism of these bacteria. Antibiotics affect cellular metabolism, but the metabolic state of the cell also influences all aspects of antibiotic use: from drug effectiveness to the development of antimicrobial resistance (2).

Most of MDR bacteria isolated from WWTPs are representatives of the fecal microbiota and include *Aeromonas* (3)*, Vibrio* (4, 5)*, Enterococcus faecium*, *Staphylococcus aureus*, *Klebsiella pneumoniae*, *Acinetobacter baumannii, Pseudomonas aeruginosa,* and *Enterobacter* spp. (ESKAPE) (6, 7), which are highly virulent and often resistant to antibiotics. Antibiotic resistance genes (ARG) and virulence genes are non-essential for survival of bacteria and are often carried by mobile genetic elements (MGEs). MGEs are inherently capable of hijacking and shuffling genes within and between cells, conferring adaptive functions on their bacterial host and altering its fitness (8).The diversity of MDR bacteria isolated from WWTPs is expanding, and a vivid example of such MDR bacteria are members of the genus *Citrobacter* (*Gammaproteobacteria; Enterobacterales; Enterobacteriaceae*). Members of the genus *Citrobacter* are facultative anaerobic chemoorganotrophic organisms (9). *Citrobacter* species are widespread and have been found in water systems, soils, food, and in humans and animals (10). Among the *Citrobacter* species*, Citrobacter freundii*, *Citrobacter koseri,* and *Citrobacter braakii* are well-known opportunistic human pathogens that cause a number of diseases. The list of diseases caused by bacteria of the genus *Citrobacter* is quite extensive: diarrheal diseases, urinary and respiratory tract infections, wound and blood infections (*C. freundii*), peritonitis and food poisoning (*C. braakii*), as well as meningitis and brain abscess in newborns and immunocompromised individuals (*C. koseri*) (10–12).

In recent years, MDR *Citrobacter* strains have been actively isolated from WWTP-associated samples, and their genetic organization and resistome have been studied. The interest in *Citrobacter* is due to the epidemiological significance of these bacteria, as they may contain critical mobile antibiotic resistance genes. For instance, *Citrobacter* strains isolated from WWTP in Australia carried multiple ARG, including colistin resistance genes (*mcr-9*) (12). Most *Citrobacter* strains isolated from wastewater produced carbapenemase and extended-spectrum β-lactamase (ESBL), and these ARGs were associated with a wide spectrum of MGEs (13–15).

*Citrobacter* virulence factors are also diverse and species-specific (12). Biofilm formation is an important process for most opportunistic pathogens, enabling bacteria to survive harsh conditions, including antibiotic treatment. Biofilm components can include polysaccharides, proteins, and even extracellular DNA (16). Analysis of extracellular polysaccharides of bacterial biofilm matrices showed that a number of carbohydrates, including cellulose, as well as D-glucose, D-galactose, D-mannose, and other sugars, are involved in the formation of extracellular polymeric substances (EPS) (17). In particular, cellulose is produced by many bacteria to serve as a biofilm matrix polymer (18, 19). The genes responsible for EPS production are usually clustered on the chromosome or on large plasmids (20).

Here, we report the isolation and genome analysis of the MDR strain of *Citrobacter portucalensis* from a Moscow WWTPs. This strain contained five circular plasmids carrying various advantageous “accessory” determinants, including genes involved in EPS formation, glycerol dissimilation, resistance to toxic substances, and ARGs.

## MATERIALS AND METHODS

### Pure culture isolation and characterization

A sample of wastewater that had undergone primary physical treatment (before microbiological treatment in bioreactors) was collected at the Kuryanovsky WWTP complex of Mosvodokanal JSC in August 2024. Serial dilutions of the water sample were plated on LB agar medium supplemented with tetracycline, chloramphenicol, kanamycin, and ampicillin at concentrations of 20 µg/mL, 20 µg/mL, 50 µg/mL, 50 µg/mL, respectively. The plates were incubated at 20°C for 3–5 days, and then individual colonies were picked up and grown in liquid LB medium with the same antibiotics. Cells were collected by centrifugation at 3,000 *g* for 10 min at 4°C. Genomic DNA was extracted using the Power Soil DNA isolation kit (Qiagen, Germany). The amount of genomic DNA was determined using the Qubit dsDNA HS Assay Kit (ThermoFisher).

The purity of the cultures was determined through microscopic examination of bacterial morphology and genome sequencing. The 16S rRNA gene was amplified using the primer pairs 27F and 1492R (21) and sequenced using the Sanger method. Preliminary taxonomic identification of the strains was performed using a BLASTN search against the NCBI GenBank database. One strain, designated KOS1-1 and assigned to *Citrobacter* sp., was further characterized.

The morphology of KOS1-1 cells was examined using transmission electron microscopy (TEM). Cells were placed on copper grids and stained with 2% (wt/vol) phosphotungstic acid. TEM images acquired on a JEM-1400 instrument operated at 80 kV at a magnification of 25,000 ×.

To determine the susceptibility/resistance of the KOS1-1 strain to antibiotics, mercury, and arsenic salts, bacteria were grown in liquid LB medium overnight at 20°C and then streaked onto LB plates supplemented with various concentrations of the substances being studied. The plates were further incubated at 20°C for 3 days. The minimum inhibitory concentration (MIC) was defined as the lowest concentration that prevented visible bacterial growth. The following substances were used: ampicillin, kanamycin, tetracycline, chloramphenicol, cefazolin, cefaclor, cefatrizine, ciprofloxacin, erythromycin, streptomycin, spectinomycin, sulfamethoxazole, trimethoprim, colistin, NaAsO_2_ [As(III)], Na_2_HAsO_4_ [As(V)], and HgCl_2_.

### Genome sequencing and analysis

Genomic DNA of strain KOS1-1 was sequenced using Illumina and Oxford Nanopore technologies. For Illumina sequencing, the library was prepared using NEBNext Ultra II DNA Library prep kit (New England Biolabs, Ipswich, MA, USA) and sequenced on an Illumina MiSeq instrument in paired-end read format (2×300 nt). A total of 741,626 read pairs (0.44 Gb) was generated. Paired reads were combined using FLASH v.1.2.11 (22). Adapter removal and exclusion of low-quality sequences (Q<30) were performed using Cutadapt v.1.8.3 (23) and Sickle v.1.33 (https://github.com/najoshi/sickle), respectively.

Genomic DNA was additionally sequenced on a MinION instrument (Oxford Nanopore Technologies, Oxford, UK) using the 1D Genomic DNA ligation sequencing kit LSK-109 and FLO-MIN106D cell. A total of 276 Mb (17,354 reads with an average length of 15,880 nt) were obtained. The MinION reads were assembled using Flye v. 2.9.5 (24) with --nano-hq and --single parameters. The sequences of the assembled contigs were polished with Illumina reads using Pypolca v. 0.3.1 (25).

The assembled genome of strain KOS1-1 was taxonomically classified using the Genome Taxonomy Database Toolkit (GTDB-Tk) v.2.4.0 (26) against the Genome Taxonomy Database (GTDB) (27). Gene search and annotation were carried out using the NCBI Prokaryotic Genome Annotation Pipeline (28) and the RAST server 2 (29). Average nucleotide sequence identity (ANI) between genomes was determined using the ani.rb script from the enveomics collection (30). IS elements in the genome were identified via BLASTN search against the ISFinder database (*E*-value ≤1 × 10⁻⁵⁰) (31). Matches covering more than 70% of the reference length were retained. Integrons were predicted using IntegronFinder v.2.0 (32). The circular maps of plasmids were generated using software Proksee Assemble v.2.0.0 (33).

Plasmid sequences were compared against the NCBI RefSeq plasmid database using BLASTN searches (*E*-value cutoff 1 × 10⁻⁵⁰, identity >80%), and hits with the highest query coverage among the top 10 hits were selected and used to calculate the ANI.

### ARG identification

Open reading frames (ORFs) were predicted in assembled contigs using Prodigal v.2.6.333 (34). ARGs were identified using the NCBI AMRFinderPlus v.4.0.5 (35) command line tool and its associated database. The predicted protein sequences of all ORFs were analyzed in this tool with the “-p” parameter.

### Production and visualization of bacterial cellulose

To obtain bacterial cellulose (BC), the KOS1-1 strain was cultured for 2 days in LB supplemented with 10 g/L glucose at 25°C with shaking. The BC pellicles were harvested by centrifugation (4,000 *g* for 15 min) and washed with 0.2 M NaOH at 80°C for 2 h to remove the medium and bacteria. Then, the samples were washed with distilled water at 80°C for 30 min three times until reaching neutral pH, resulting in a pure BC precipitate.

The morphology of BC particles was studied using atomic force microscopy (AFM) and transmission electron microscopy (TEM). Samples were placed on copper grids and stained with 2% (wt/vol) phosphotungstic acid. TEM images were taken on a JEM-1400 instrument at 10,000 × magnification. Atomic force microscopy was performed using an Integra Prima microscope and Nova SPM v.4.0 software (NT-MDT, Moscow, Russia). Scanning was performed in semi-contact mode using an NSG01 gold cantilever (NT-MDT). Particle characteristics were determined using the NT-MDT Nova v. 1.06.26 software, supplied with the instrument.

## RESULTS

### Isolation of *Citrobacter portucalensis* KOS1-1

**Fig 1:**
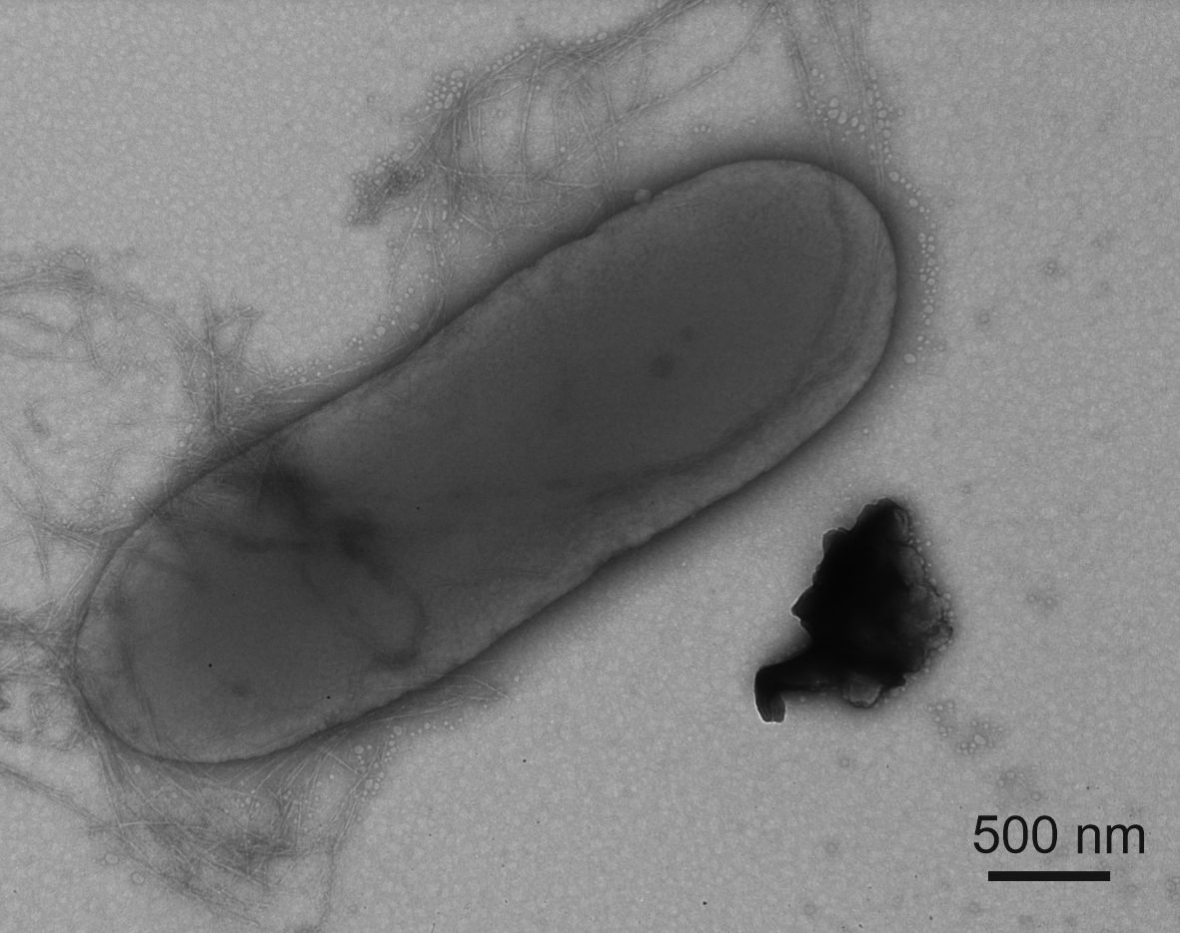
TEM micrograph of strain KOS1-1 cells.

Plating of wastewater samples on LB agar supplemented with tetracycline, kanamycin, chloramphenicol, and ampicillin showed that the titer of cultured strains resistant to all four drugs was approximately 1.1 × 10^3^ cells/mL. One of the obtained strains, KOS1-1, was assigned to the genus *Citrobacter* based on 16S rRNA gene sequencing data. The cells of strain KOS1-1 were rod shaped, approximately 3–3.5 μm long and approximately 1 μm wide (Fig. 1).

The results of the antibiotic susceptibility testing showed that the KOS1-1 strain exhibited resistance not only to tetracycline, kanamycin, chloramphenicol, and ampicillin but also to cefazolin, cefaclor, cefatrizine, ciprofloxacin, erythromycin, streptomycin, spectinomycin, and trimethoprim/sulfamethoxazole, but was susceptible to colistin (Table 1). The strain tolerated up to 25 mM and 100 mM of arsenite and arsenate, respectively, as well as up to 0.1 mM of mercury.

**TABLE 1:** MIC values of antibiotics for *C. portucalensis* KOS1-1

Class of antibiotics	Antibiotic	MIC (mg/L)
Beta-lactam	Ampicillin	>2,000
	Cefazolin	32
	Cefaclor	16
	Cefatrizine	40
Fluoroquinolone	Ciprofloxacin	4
Aminoglycoside	Streptomycin	500
	Spectinomycin	1,000
	Kanamycin	>2,000
Macrolide	Erythromycin	>1,000
Phenicol	Chloramphenicol	500
Tetracycline	Tetracycline	>500
Diaminopyrimidine/sulfonamide	Trimethoprim/sulfamethoxazole	>160/800
Polymyxin	Colistin	<4

### Genome of *Citrobacter portucalensis* KOS1-1

Sequencing and assembly of the KOS1-1 strain genome yielded six circular contigs representing the chromosome and five plasmids (Table 2). Taxonomic placement of the KOS1-1 genome in the GTDB showed that this strain belongs to the species *Citrobacter portucalensis*. The ANI value between the KOS1-1 genome and the genome of the type strain *C. portucalensis* A60^T^ (36) was 98.28%, which again confirms that the KOS1-1 strain belongs to this species. The KOS1-1 genome contained genes encoding enzymes of key metabolic pathways of chemoorganotrophic bacteria, including glycolysis, the tricarboxylic acid cycle, the pentose–phosphate pathway, and the aerobic respiratory chain. The presence of genes encoding the respiratory nitrate reductase suggests that the KOS1-1 strain can use nitrate as an electron acceptor under anaerobic conditions and participate in nitrogen removal from wastewater, as previously suggested for the *C. portucalensis* strain AAK_AS5 (9).

**TABLE 2:** Main characteristics of *C. portucalensis* KOS1-1 genome

	Length (bp)	GC content	Copy number^*a*^	Protein-coding genes	Replicons	IS elements	ARGs	Resistance to arsenic and mercury
Chromosome	4,878,312	51.59	(1)	4,514	1	68	3	*arsRDABC*
pKOS1-1-1	77,569	47.48	1.06	75	4	20	0	
pKOS1-1-2	80,667	53.48	1.19	68	2	13	0	*arsRHCB*
pKOS1-1-3	110,147	51.03	0.87	119	1	2	1	
pKOS1-1-4	121,081	53.88	1.24	121	1	8	2	
pKOS1-1-5	289,033	48.02	0.98	330	1	32	8	*merRTPCADE; arsRDAB*

^*a*^ According to the average sequence coverage.

In addition to the chromosome, the KOS1-1 strain harbored five low copy number plasmids ranging in size from 77,569 to 289,033 bp (Table 2). A BLASTN search against the NCBI RefSeq database revealed similar plasmids among members of the *Enterobacteriaceae* family (Table S1). Although local sequence similarity was high, it was observed only for particular fragments of the plasmids (54%–87% of the total length), which is consistent with the concept of a mosaic structure of enterobacterial plasmids.

**Fig 2:**
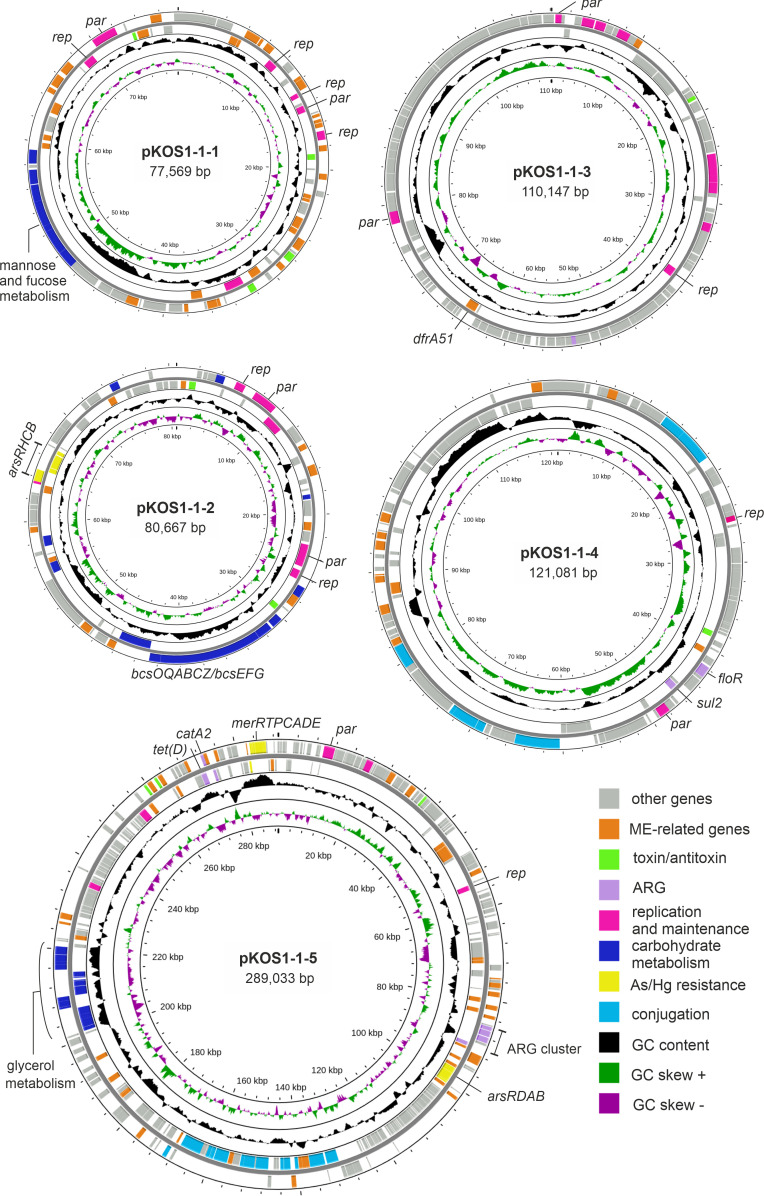
Schematic maps of plasmids identified in *C. portucalensis* strain KOS1-1. Protein-coding genes on the forward and reverse strands are shown as rectangles outside and inside the main circle, respectively. The innermost circle presents GC-skew in green (+) and purple (−), and the next-to-innermost circle represents G+C content (deviation from the average) in black (+, outward; −, inward).

The mosaic structure of the pKOS1-1-1 plasmid was further indicated by the presence of four genes encoding distantly related plasmid replication initiator proteins, two of which were associated with plasmid partitioning operons (Fig. 2). Two distinct replicons, including the replication initiation and partitioning genes, were found in plasmid pKOS1-1-2 (Fig. 2). The other three plasmids, pKOS1-1-3, pKOS1-1-4, and pKOS1-1-5, contained single replicons of the IncFIB(pHCM2), IncA/C, and pKPC-CAV1321 groups, respectively. Plasmids pKOS1-1-4 and pKOS1-1-5 contained clusters of genes encoding conjugative transfer proteins and probably could be transmitted by conjugation (Fig. 2).

Plasmid pKOS1-1-3, in addition to the replication and maintenance functions characteristic of plasmids, encoded bacteriophage-related proteins, including terminase, head, and tail structural proteins, closely related to the corresponding proteins of the *Salmonella* phage SSU5. Therefore, pKOS1-1-3 is likely a so called phage-plasmid, that is, a temperate bacteriophage that, in the lysogenic state, does not integrate into the chromosome but is maintained as a plasmid (37).

Plasmids are known to contain genes that enable the host to detoxify harmful compounds. In accordance with the observed tolerance of strain KOS1-1 to mercury, a mercury resistance gene cluster (*merRTPCADE*) was found on plasmid pKOS1-1-5 (Fig. 2). This plasmid also contained the *arsRDAB* genes enabling the export of arsenite from the cell, while the arsenate reductase gene *arsC,* together with the genes *arsH, arsR*, and *arsB,* was located on plasmid pKOS1-1-2 (Fig. 2). In addition, a complete arsenic resistance operon comprising the *arsRDABC* genes was found on the chromosome. The activity of these operons may explain the phenotypic resistance of the KOS1-1 strain to arsenate and arsenite. Close homologs of each of the three *ars* gene clusters were found on the chromosomes of *Citrobacter* species (for the chromosomal *ars* operon) and plasmids of various enterobacteria. However, the common genes of the three *ars* clusters are significantly less similar to each other. Probably, the plasmid-localized *ars* gene clusters did not arise as a result of recent transfer from the *Citrobacter* chromosome but have a different origin.

### Resistome of *C. portucalensis* KOS1-1

A search for ARGs in the *C. portucalensis* KOS1-1 genome predicted three ARGs in the chromosome and 11 ARGs in plasmids (Table 3 and Table S2). Interestingly, most of the resistance genes were associated with integrons, which are widely known for their role in the dissemination of antibiotic resistance (38). The chromosome contained the genes *qnrB, aph(3′)-I,* and *bla_CMY_* which could confer resistance to quinolones, gentamicin/kanamycin, and cephalosporins, respectively. The largest plasmid, pKOS1-1-5, contained a cluster of ARGs comprising *dfrA16, aadA2, ere(A), qacE, sul1*, and *bla_TEM-1_*, as well as the distantly located *tet(D*) and *catA2* genes. According to the NCBI AMR database, these genes could determine resistance to trimethoprim, streptomycin, erythromycin, sulfonamides, beta-lactams, tetracycline, and chloramphenicol. Plasmid pKOS1-1-3 contained the *dfrA51* gene, encoding trimethoprim-resistant dihydrofolate reductase. The genes *floR* for the chloramphenicol/florfenicol efflux MFS transporter and *sul2* for the sulfonamide-resistant dihydropteroate synthase Sul2 were found in plasmid pKOS1-1-4. The identified set of ARGs may explain the observed phenotypic resistance of the KOS1-1 strain to beta-lactams, aminoglycosides, ciprofloxacin, erythromycin, chloramphenicol, tetracycline, trimethoprim, and sulfamethoxazole (Table 1).

**TABLE 3:** ARGs detected in the *C. portucalensis* KOS1-1 genome

Location	Gene	Product	Drug class	Drug subclass
Chromosome	*bla_CMY_*	CMY-2 family class C beta-lactamase	Beta-lactam	Cephalosporin
Chromosome	*qnrB17*	Quinolone resistance pentapeptide repeat protein QnrB17	Quinolone	Quinolone
Chromosome	*aph(3′)-I*	APH(3′)-I family aminoglycoside O-phosphotransferase	Aminoglycoside	Gentamicin/ kanamycin
pKOS1-1-3	*dfrA51*	Trimethoprim-resistant dihydrofolate reductase DfrA51	Trimethoprim	Trimethoprim
pKOS1-1-4	*floR*	Chloramphenicol/florfenicol efflux MFS transporter FloR	Phenicol	Chloramphenicol/ florfenicol
pKOS1-1-4	*sul2*	Sulfonamide-resistant dihydropteroate synthase Sul2	Sulfonamide	Sulfonamide
pKOS1-1-5	*dfrA16*	Trimethoprim-resistant dihydrofolate reductase DfrA16	Trimethoprim	Trimethoprim
pKOS1-1-5	*aadA2*	ANT(3′′)-Ia family aminoglycoside nucleotidyltransferase AadA2	Aminoglycoside	Streptomycin
pKOS1-1-5	*ere(A*)	EreA family erythromycin esterase	Macrolide	Erythromycin
pKOS1-1-5	*qacE*	Quaternary ammonium compound efflux SMR transporter QacE delta 1	Quaternary ammonium compounds	Quaternary ammonium compounds
pKOS1-1-5	*sul1*	Sulfonamide-resistant dihydropteroate synthase Sul1	Sulfonamide	Sulfonamide
pKOS1-1-5	*bla_TEM-1_*	Broad-spectrum class A beta-lactamase TEM-1	Beta-lactam	Beta-lactam
pKOS1-1-5	*tet(D*)	Tetracycline efflux MFS transporter Tet(D)	Tetracycline	Tetracycline
pKOS1-1-5	*catA2*	Type A-2 chloramphenicol O- acetyltransferase CatII	Phenicol	Chloramphenicol

### Mobile genetic elements

The chromosome of strain KOS1-1 contained 68 copies of IS elements representing different families (Table S3), none of which contained ARGs. The most numerous were IS*1*R elements found in 32 regions of the chromosome.

**Fig 3:**
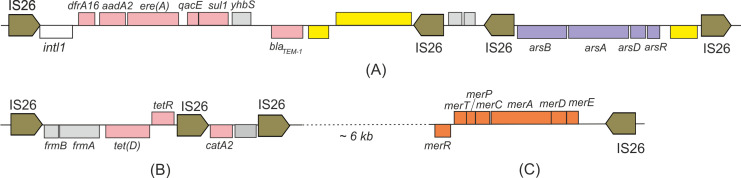
Clusters of genes for resistance to antibiotics, arsenic, and mercury in the pKOS1-1-5 plasmid. Rectangles immediately above and below the main line represent predicted genes that are transcribed rightward and leftward, respectively; their colors indicate functional classification as follows: ARGs (pink), transposases/resolvases (yellow), arsenate resistance (violet), mercury resistance (orange), and others (gray). The truncated integron integrase gene *intl1* is shown as an open rectangle. Mobile elements are shown by arrows. The coordinates of the shown plasmid regions according to the GenBank sequence CP199071: (**A**) 268847–276116 (**B**), and 282463–288000 (**C**).

In contrast to the chromosome, a variety of IS elements were found in plasmids, the most abundant of which were IS*26*, IS*1*R, and ISKpn*26*. The conjugative plasmid pKOS1-1-5 was particularly rich in mobile elements. It harbored an about 10 kb long region containing an ARG cassette including genes *dfrA16, aadA2, ere(A), qacE, sul1,* and *bla_TEM-1_*, along with the Tn*3* family transposase and recombinase genes. The first three ARGs were predicted to be associated with class 1 integron; however, its integrase gene was truncated by the insertion of the IS*26* element into its 3′- region (Fig. 3), likely rendering it no longer functional. This gene cluster was flanked by inverted copies of the IS*26* element (Fig. 3). A structurally similar cluster located between two inverted copies of the IS*26* element comprised genes *arsRDAB* of the arsenic resistance operon (Fig. 3). Another gene island contained three direct copies of IS*26* flanking the *tet(D*) gene encoding a tetracycline efflux transporter with the *tetR* regulatory gene, the chloramphenicol O-acetyltransferase type A-2 gene (*catA2*), as well as the S-formylglutathione hydrolase *(frmB)* and S-(hydroxymethyl)glutathione dehydrogenase *(frmA)* genes (Fig. 3).

### Gene clusters mediating carbon metabolism located on plasmids

Bacteria utilize diverse carbon sources not only to generate energy and build cellular compounds but also for biofilm formation. Polysaccharides, key components of the biofilm matrix, have been shown to contribute to the virulence of pathogenic bacteria (39). Although genes involved in carbon metabolism are typically localized on chromosomes, in strain KOS1-1 several clusters of such genes were found on plasmids.

Plasmid pKOS1-1-1 contained a cluster of genes encoding GDP-mannose 4,6-dehydratase, GDP-L-fucose synthetase, GDP-mannose-mannosylhydrolase, mannose-1-phosphate guanylyltransferase/mannose-6-phosphate isomerase, glycosyltransferase family 2 (GT2), phosphomannomutase, and mannose-6-phosphate isomerase. These enzymes can enable the synthesis of GDP-mannose and GDP-fucose from fructose-6-phosphate, an intermediate product of glycolysis. GDP-mannose acts as a mannose donor for the building of cell wall polymers and other mannose-containing glycoconjugates, while GDP-fucose is crucial for fucose-containing exopolysaccharides. This genomic island (Fig. 2) was 7,434 bp in length and exhibited 84.5% nucleotide sequence identity with a region on a plasmid from *Proteus terrae* subsp. cibaricus strain SDQ8C180-2T (CP073357.1). It also showed 84.23% identity with two *Vibrio alginolyticus* chromosomal contigs (CP076287.1 and CP016224.1) and 83.37% identity with the chromosome of *Vibrio natriegens* (CP129942.1). Interestingly, homologs of most of these genes were also found in the chromosome of strain KOS1-1 where they are included in a larger colanic acid biosynthesis gene cluster. Colanic acid is composed of polyanionic heteropolysaccharides with hexasaccharide repeating units, consisting of glucose, fucose, galactose, and glucuronic acid. It is excreted by the cell to form a protective bacterial capsule and plays a role in biofilm formation (40). However, the nucleotide sequence identity between the chromosomal and plasmid copies of these genes was less than 70%, indicating that the plasmid genes originated from other bacterial species.

**Fig 4:**
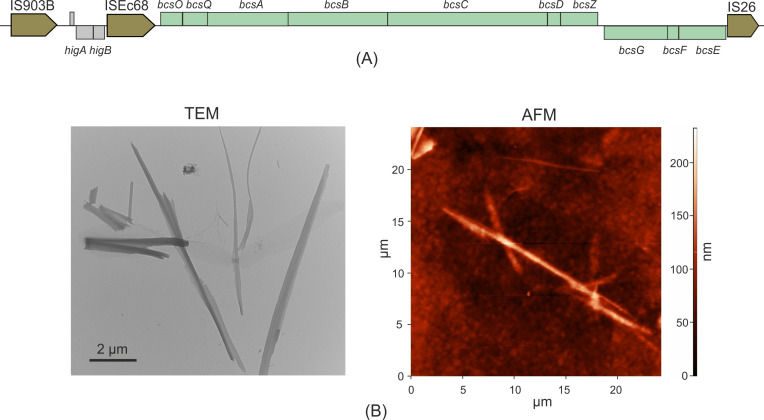
Production of bacterial cellulose. (**A**) Bacterial cellulose synthase gene cluster in plasmid pKOS1-1-2. Rectangles immediately above and below the main line represent predicted genes that are transcribed rightward and leftward, respectively; their colors indicate functional classification as follows: *bcs-*related genes (green); others (gray). Mobile elements are shown by arrows. (**B**) TEM and AFM images of cellulose produced by pKOS1-1 strain.

Plasmid pKOS1-1-2 contained a 19 kb long region, including genes for the HigA/HigB type II toxin-antitoxin system, and the bacterial cellulose synthase (*bcs*) gene cluster *bcsOQABCZ/bcsEFG* which could enable the biosynthesis of bacterial cellulose which forms protective envelopes around the cells (19). This genomic island contained the IS elements IS*903*B and IS*26* which flank its boundaries, and ISEc*68*, located between the *higA/higB* genes and the *bcs* gene cluster (Fig. 4A). BLASTN analysis revealed that this genomic island shared 99.6% nucleotide sequence identity with the homologous region in the *Citrobacter freundii* plasmid pRHBSTW-00915 (CP056246.1). The plasmid *bcs* cluster is the only one in the KOS-1-1 strain; homologs of the *bcs* genes were not found in the chromosome.

Transmission electron microscopy and atomic force microscopy methods were used to visualize bacterial cellulose (Fig. 4B). Both methods confirmed the presence of rod-shaped nanofibers in the culture of strain KOS-1-1. The diameter of the nanofibers was about 80–150 nm while their length reached several tens of micrometers. Similar morphology of BC fibers has been reported in other studies (41–43).

**Fig 5:**
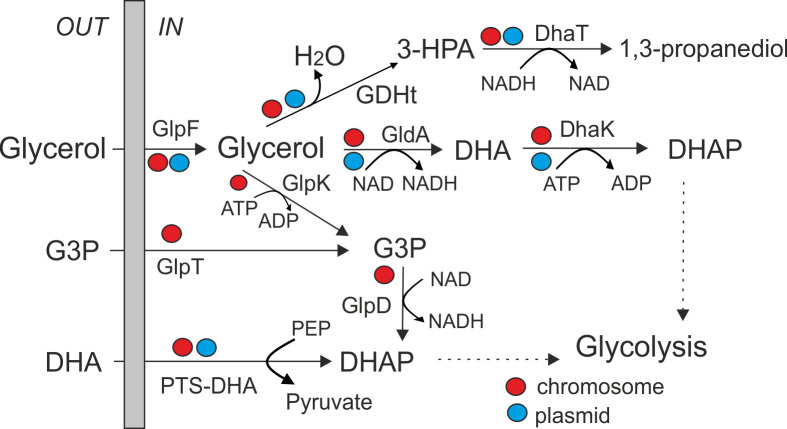
Metabolic pathways for glycerol utilization in the KOS-1-1 strain. Abbreviations: DHA, dihydroxyacetone; DHAP, dihydroxyacetone phosphate; G3P, glycerol-3-phosphate; PEP, phosphoenolpyruvate; GlpF, glycerol uptake facilitator; GDHt, glycerol dehydratase; DhaT, 1,3-propanediol dehydrogenase; GldA, glycerol dehydrogenase; DhaK, ATP-dependent dihydroxyacetone kinase; GlpK, glycerol kinase; GlpT, glycerol-3-phosphate transporter; GlpD, aerobic sn-glycerol-3-phosphate dehydrogenase; PTS-DHA, phosphoenolpyruvate-dihydroxyacetone phosphotransferase system. Genes located in the chromosome and plasmid pKOS1-1-5 are marked with red and blue circles, respectively.

Glycerol is one of the carbon sources that can be utilized by bacteria. It is also involved in the biosynthesis of phospholipids, along with dihydroxyacetone phosphate (DHAP), an intermediate of glycolysis. Microorganisms can produce glycerol 3-phosphate (G3P) from endogenous DHAP and/or exogenous glycerol to synthesize glycerophospholipids from G3P (44). Plasmid pKOS1-1-5 contained a large (~19 kb) cluster of genes involved in glycerol uptake and metabolism (Fig. 2). Close homologs of this cluster were found in the chromosomes and plasmids of bacteria of the genera *Salmonella, Kosakonia, Klebsiella, Enterobacter*, and *Citrobacter*. This cluster encodes the glycerol uptake facilitator (GlpF), glycerol dehydrogenase (GldA), which converts glycerol to dihydroxyacetone (DHA), and dihydroxyacetone kinase, which phosphorylates DHA to DHAP in an ATP-dependent manner (Fig. 5). Alternatively, DHA can be imported and phosphorylated by the phosphoenolpyruvate-dihydroxyacetone phosphotransferase system (PTS-DHA, genes *dhaKLM*), encoded within the same locus. This process is regulated by the transcriptional regulator DhaR. Glycerol is also the main substrate in the 1,3-propanediol (1,3-PDO) biosynthetic pathway. In this pathway, glycerol dehydratase converts glycerol into 3-hydroxypropionaldehyde (3-HPA). This reaction requires adenosylcobalamin (coenzyme B12), synthesized by ATP:Cob(I)alamin adenosyltransferase. Two additional genes encode the glycerol dehydratase reactivation factor (large and small subunits). In the final step, 1,3-propanediol dehydrogenase (DhaT) reduces 3-HPA to 1,3-propanediol (1,3-PDO) with concomitant re-oxidation of NADH. Close homologs of this glycerol utilization cluster have been found on different plasmids (e.g., plasmid pF321-1 of *C. portucalensis* strain F321), and more distant homologs of all genes within the described cluster are also present in the chromosome of strain KOS1-1, although they are dispersed across three loci.

## DISCUSSION

*Citrobacter* species are opportunistic pathogens found in humans, animals, and various environments, including soil and water. However, the genomic characteristics of plasmids and other mobile genetic elements of *Citrobacter*, particularly with regard to antimicrobial resistance, remain poorly understood. The aim of this study was to analyze the genome of the MDR *Citrobacter portucalensis* strain KOS1-1 isolated from wastewater, focusing on its plasmids and their beneficial adaptive properties. As noted previously, members of the genus *Citrobacter* were found in diverse environments, with wastewater samples representing a high-risk reservoir. The species *C. portucalensis* was proposed in 2017 for strain A60^T^, isolated from a well water sample in Portugal (36). *C. portucalensis* strains found in diverse environments worldwide contained different sets of clinically significant genes conferring resistance to carbapenems, broad-spectrum cephalosporins, cephamycins, aminoglycosides, fluoroquinolones, disinfectants, and heavy metals (45). Between 2016 and 2023, 114 carbapenem-resistant *Citrobacter* strains were isolated from hospital wastewater in several provinces of China (46). An additional 60 MDR *Citrobacter* strains were isolated from Australian wastewater treatment plants (12). Notably, of the approximately 2,000 high-quality *Citrobacter* genomes in the GTDB, 138 were obtained from WWTP-related samples. *Citrobacter* is an emerging pathogen with multidrug resistance in hospitalized patients (47, 48). The high prevalence of these bacteria in Moscow hospitals (49, 50) suggests that the source of our KOS1-1 strain isolated from influent wastewater may be of hospital origin.

Initially, we isolated this strain on a selective medium containing tetracycline, chloramphenicol, kanamycin, and ampicillin to target MDR bacteria. Genome analysis revealed ARGs associated with resistance to these drugs: *tet(D)* (tetracycline), *catA2* and *floR* (chloramphenicol), *aph(3′)-Ia* and *aadA2* (kanamycin/ aminoglycosides), *bla_CMY_* (cephalosporins), and *bla_TEM-1_* (beta-lactams), which likely explain the observed phenotypic traits. The genome of the strain KOS1-1 also contains ARGs for other drugs, such as sulfonamide (*sul1* and *sul2*), quinolones (*qnrB*), erythromycin (*ere(A*)), and trimethoprim (*dfrA51*). Consistently, it was demonstrated that the strain is resistant to antibiotics of these classes. A comparative analysis of 71 *C*. *portucalensis* genomes available in public databases (45) showed that the chromosomal genes *bla_CMY_* and *qnrB* were present in almost all genomes, whereas other ARGs were found only in some strains, with ARGs to sulfonamides and phenicols being particularly rare, and *ere(A*) being absent. Overall, it can be concluded that *Citrobacter* species frequently harbor various different resistance determinants, with particularly high resistance rates observed for third-generation cephalosporins, gentamicin, and fluoroquinolones (48).

Mobile genetic elements, such as insertion sequences and transposons, are capable of moving within or between DNA molecules and play a central role in horizontal gene transfer, thereby promoting the acquisition and dissemination of resistance genes (51). The KOS1-1 strain harbors several IS elements on its plasmids, which may facilitate the dissemination of ARGs. A striking example is the long TnAs*3*-type element found on plasmid pKOS1-1-5, which contain several ARGs (*dfrA16, aadA2, ere(A), qacE, sul1,* and *bla_TEM-1_*) and is flanked by inverted copies of the IS*26* element. In *Salmonella*, TnAs*3* has previously been reported to be associated with the *ant(3)-Ia, dfrA15, qacE*, and *sul1* genes (52). In the *Morganella morganii* plasmid, the integron containing the *aadA7* and *sul1* genes was bracketed by the composite transposon TnAs*3* (53). Studies of clinical *C. freundii* isolates in China revealed that the ARG cassette was located on TnAs*3* being flanked by IS*91* elements (54).

The second ARG-containing island located on the plasmid pKOS1-1-5 has the structure IS*26-frmB-frmA-tet(D)-tetR*-IS*26-catA2-hyp*-IS*26*. This plasmid also carried an arsenic resistance gene cluster (*arsRDAB*) flanked by inverted copies of the IS*26* element. IS*26* family mobile elements play an important role in the dissemination of antibiotic resistance determinants and heavy metal resistance genes in Gram-negative bacteria (55–57). IS*26* elements were frequently found within pseudo-composite transposons, which mobilize diverse variants of the β-lactamase gene and the sulfonamide resistance gene *sul1* (58). The ARGs found in strain KOS1-1, associated with IS*26* elements, may also serve as examples of pseudo-composite transposons. Similar data were reported by Harmer and Hall (57).

Plasmids pKOS1-1-4 and pKOS1-1-5 contained *tra* gene clusters which may facilitate their conjugative transfer. Such plasmids can integrate a wide range of ARGs, as well as virulence-related genes (59), which, combined with their ability to perform conjugative transfer, makes them an important factor in the spread of various resistance genes. Plasmid pKOS1-1-5 contained two clusters of ARGs, arsenic and mercury resistance gene clusters, and may play such a role in wastewater treatment plant environments.

Biofilms are structured communities of microorganisms that adhere to surfaces and play a key role in maintaining the persistence of bacterial infections (60). Cellulose is a key structural component of the biofilm matrix, providing both mechanical stability and protection for microbial communities. Plasmid pKOS1-1-2 contained the complete *bcsOQABCDZGFE* operon responsible for cellulose biosynthesis. This operon is likely located within a pseudo-composite transposon, as it is flanked by the IS*903*B and IS*26* insertion sequences. A homology search of the pKOS1-1-2 *bcsOQABCDZGFE* operon against the NCBI GenBank database, along with numerous chromosomal homologs, revealed 20 plasmids containing the majority of this operon (>99% identity, >86% query coverage) and hosted by *Shigella flexneri*, *Klebsiella variicola, Escherichia coli, Raoultella ornithinolytica*, and *Klebsiella pneumoniae*. To the best of our knowledge, this is the first report demonstrating that the *bcs* cellulose biosynthesis operon can be located not only on the chromosome but also on plasmids that could facilitate its horizontal transfer potentially enhancing adaptive capabilities of bacteria.

Another genetic region located on the pKOS1-1-1 plasmid includes genes involved in GDP-mannose and GDP-fucose metabolism and may also be associated with biofilm formation. Previous experimental evidence has shown that mannose-6-phosphate isomerase and GDP-mannose 4,6-dehydratase in *Azospirillum brasilense* Sp7 contributed to the biosynthesis of lipopolysaccharides and exopolysaccharides, which are key components of biofilm development (61). The role of the phosphomannose isomerase gene (*manA*) in biofilm formation has also been documented in *Photorhabdus luminescens* (62). The genomic island identified in this study has close homologs in the plasmids and chromosomes of gamma-proteobacteria. Interestingly, the chromosome of strain KOS1-1 contains homologous genes within the colanic acid biosynthesis gene cluster. However, nucleotide sequence identity between the chromosomal and plasmid copies of these genes is quite low, suggesting the acquisition of plasmid genes from other bacterial species. Perhaps their presence in the KOS1-1 strain provides modifications of the components of the biofilm matrix.

A distinct genomic island associated with glycerol uptake and metabolic transformations was identified in the pKOS1-1-5 plasmid. A search of the NCBI database revealed more than fifty highly similar sequences across plasmids and chromosomes of multiple bacterial genera, including *Salmonella, Kosakonia, Klebsiella, Enterobacter*, and *Citrobacter*, suggesting that this region has likely been disseminated through horizontal gene transfer. The ability to metabolize glycerol enhances biofilm formation in *Pseudomonas aeruginosa*, and the deletion of the glycerol metabolism repressor *glpR* further enhances biofilm formation by upregulating the synthesis of the polysaccharide Pel (63). Glycerol metabolism also promotes biofilm formation in other bacterial species. For example, in *Bacillus subtilis*, glycerol in combination with manganese stimulates biofilm formation via histidine kinase signaling (64). Numerous studies have shown that a key enzyme in glycerol metabolism, glycerol-3-phosphate dehydrogenase, is involved in biofilm formation (65–67). The potential of a plasmid-located gene cluster associated with glycerol uptake and its metabolic transformations for modulating biofilm formation in the KOS1-1 strain warrants further studies.

Of the adaptive genomic loci absent from the chromosome of strain pKOS1-1, clusters of ARGs are localized on plasmid pKOS1-1-5, one ARG on pKOS1-1-3, and two ARGs on pKOS1-1-3. Furthermore, plasmid pKOS1-1-5 carries a cluster of mercury resistance genes, and pKOS1-1-2 contains the *bcs* operon, responsible for cellulose biosynthesis and involved in biofilm formation. All these functions can be used by bacteria under the pressure of antibiotics and other toxic compounds which contribute to the positive selection of plasmid-containing strains.

At the same time, a number of potentially adaptive functions are duplicated on plasmids and the chromosome although this does not result from a recent transfer of chromosomal genes to plasmids. Thus, in addition to a complete chromosomal copy of the arsenic resistance operon, its individual genes are present on plasmids. Along with chromosomal copies, there are also plasmid-carried genes involved in glycerol, fructose, and mannose metabolism. It remains unclear to what extent such functional redundancy may be beneficial to the bacterium. It is possible that the enzymes encoded by additional copies of the *ars* genes have different characteristics (e.g., substrate affinity), which expands the range of its functioning. Since enzymes involved in glycerol, fructose, and mannose metabolism can influence the formation of the polysaccharide matrix of biofilms, plasmid copies of these genes may also provide adaptive advantages under certain growth conditions.

### Conclusions

This study provides the first characterization of resistome and mobilome of the multidrug-resistant *Citrobacter portucalensis* KOS1-1 strain isolated from a wastewater treatment plant. Most of the ARGs and heavy metal resistance genes were located on plasmids, where many of them were associated with putative pseudo-composite transposons. The plasmids contain the complete cellulose biosynthesis gene cluster, as well as genes involved in mannose and fucose metabolism, presumably involved in biofilm formation, as well as genes for glycerol metabolism. Acquisition of these plasmid-harbored genomic islands may enhance bacterial adaptability and competitiveness. The presence of these metabolic and biofilm-associated gene clusters suggests a role in the survival of *C. portucalensis* KOS1-1 under harsh environmental conditions and may contribute to virulence.

## Data Availability

The complete genome sequence of *Citrobacter portucalensis* strain KOS1-1 has been deposited in the NCBI GenBank database under the accession numbers CP199069–CP199074.

## SUPPLEMENTAL MATERIAL

Supplemental tables spectrum.02038-25-s0001.xlsx Tables S1 to S3.

ASM does not own the copyrights to Supplemental Material that may be linked to, or accessed through, an article. The authors have granted ASM a non-exclusive, world-wide license to publish the Supplemental Material files. Please contact the corresponding author directly for reuse.
